# Chemical Components and Cardiovascular Activities of *Valeriana* spp.

**DOI:** 10.1155/2015/947619

**Published:** 2015-12-16

**Authors:** Heng-Wen Chen, Ben-Jun Wei, Xuan-Hui He, Yan Liu, Jie Wang

**Affiliations:** ^1^Guang'anmen Hospital, China Academy of Chinese Medical Sciences, Beijing 100053, China; ^2^Hubei University of Traditional Chinese Medicine, Wuhan, Hubei 430062, China; ^3^Department of Pharmaceutical Chemistry, Beijing Institute of Radiation Medicine, Beijing 100850, China; ^4^Key Laboratory of Chinese Materia Medica, Heilongjiang University of Chinese Medicine, Ministry of Education, Harbin, Heilongjiang 150036, China

## Abstract

*Valeriana* spp. is a flowering plant that is well known for its essential oils, iridoid compounds such as monoterpenes and sesquiterpenes, flavonoids, alkaloids, amino acids, and lignanoids.* Valeriana* spp. exhibits a wide range of biological activities such as lowering blood pressure and heart rate, antimyocardial ischemia reperfusion injury, antiarrhythmia, and regulation of blood lipid levels. This review focuses on the chemical constituents and cardiovascular activities of* Valeriana* spp.

## 1. Introduction

*Valeriana officinalis* Linn, perennial herbaceous plant belonging to the Valerianaceae family, is widely distributed in temperate regions. It comprises approximately 250 species, and 11 out of the 28 (including 1 variant) Chinese varieties are used as herbal medicines [1–4]. Most research studies have focused on six species:* V. officinalis* L.,* V. jatamansi* Jones,* V. officinalis* L. var.* latifolia* Miq.,* V. amurensis* Smir. ex Kom.,* V. fauriei* Briq., and* V. alternifolia* var.* stolonifera* Bar. et Skv.

The roots and rhizomes of* Valeriana* spp. are rich in essential oils, iridoids, flavonoids, alkaloids, amino acids, and lignanoids [5–9], which possess characteristic fragrance or off-flavor and are used as medicines based on their inherent bioactivities that include inducing sedation, promoting sleep, antidepression, and antianxiety [10–18].* Valeriana* spp. is now listed in the European and USA pharmacopeias. It is also sold as a diet supplement in the USA and is one of the highest selling natural medicines in Europe and the USA [19]. In addition,* Valeriana* spp. is of high medical and economic value in the food, drink, and cosmetic industries due to its distinct flavor, and current research efforts are aimed at further exploiting other features of the plant [20, 21]. This review focuses on the chemical constituents and cardiovascular activities of* Valeriana* spp., aiming at providing a theoretical foundation for further research and evaluation of its medicinal value.

## 2. Chemical Constituents

### 2.1. Essential Oils

Approximately 0.5%–2.0% of* Valeriana* spp. consists of essential oils by gas chromatography-mass spectrometry (GC-MS), which varies with species, climate, and growing environment. Valerian plants from high-altitude fertile and sandy soil have significantly higher essential oil content and yield similar to that of biennials compared to annuals. Valerian plants that produce a higher amount of essential oil are cultivated between September and November, although the content of essential oils decreases with longer periods of propagation.

A total of 150 compounds have been identified in the essential oils of Valerian plants, mainly including monoterpenes and sesquiterpenes. Most monoterpenes, namely, borneol, bornyl acetate, and isobornyl acetate, exhibit various bioactivities. Around 30 sesquiterpenes have also been detected in the Valerian essential oils. These have been classified to be of the guaiane type and valerian type. Despite the low contents of these essential oils, their biological activities have drawn the attention of researchers around the world [22–24].

**Figure 1:**
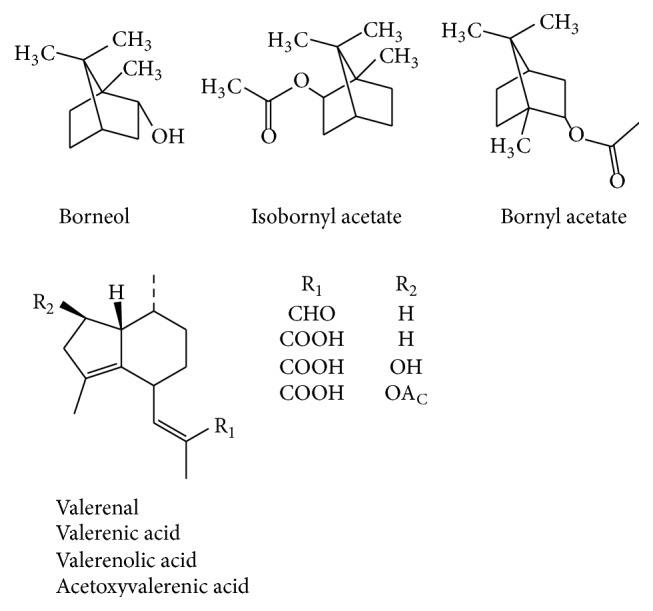
Major essential oil constituents from* V. officinalis* L. var.* latifolia* Miq.

Long et al. [25], Ming et al. [26], Wang et al. [27], and Yu et al. [28] previously investigated essential oils from* Valeriana* by GC-MS, showing its content was 1%, and 20%–60% of it was bornyl acetate. Wang et al. [27] detected 34 compounds by GC-MS, which comprised 91.75% of the total content of the essential oil of* V. officinalis* L. var.* latifolia* Miq. (Table 1 and Figure 1). Compared to the standard spectrum, bornyl acetate showed the highest content level at 23.93%, followed by nootkatone (14.79%) and 6-isopropyl-1-methyl bicycles [3,1,0] hexane (14.19%).

Yu et al. [28] analyzed essential oils from cultivated* V. officinalis* L. var.* latifolia* Miq. by GC-MS and identified 6 compounds, bornyl acetate (60.19%), (−)-acetic acid* Rhodomyrtus* enol ester (3.87%), *α*-terpinyl acetate (1.55%), acetyl carene (1.68%), *α*-selinene (26.07%), and (*Z,E*)-*α*-farnesene (1.56%), comprising 94.92% of the total content. Cultivated* V. officinalis* L. var.* latifolia* Miq. consisted of a higher number of simple components, which was predominated by bornyl acetate relative to that of wild* V. officinalis* L.

### 2.2. Iridoids

Valepotriate was first isolated from* V. wallichii* and preliminary studies by Thies and Funke [29] have confirmed the presence of a sedation ingredient. The study drew the attention of researchers from around the world. To date, over 130 iridoids from* Valeriana* spp. have been identified, possibly contributing their sedative, antidepressant, and antitumor activities.

Chen et al. [30] studied the levels of valepotriate, dihydrovalepotriate, and acetyl-valepotriate from* V. jatamansi* Jones,* V. officinalis* L., and* V. officinalis* L. var.* latifolia* Miq. by using the reverse phase high-performance liquid chromatography (RP-HPLC) method. The highest levels were observed in* V. jatamansi* Jones, followed by* V. officinalis* L. and* V. officinalis* L. var.* latifolia* Miq. In addition, the content of iridoid varied significantly among different parts and habitats.

The main iridoids in* Valeriana* comprised didrovaltrate and valepotriates derivatives (0.5%–9.0%), including valepotriate, isovalepotriate, acetoxyvalepotriate, and isovalemxy-hydroxy-dihydrovatrate. These were characterized by a hemiacetal fragment, which leads to the decomposed productions of isopentoic acid and valerienal at a specific pH or 60°C. C-1, C-7, C-10, or C-11 of compounds were mainly substituted by acyl groups such as acetyl, isovaleryl, *α*-acetoxyisovaleryl, *β*-acetoxyisovaleryl, and *β*-hydroxyisovaleryl. Furthermore, iridoids could be further divided into diethenoid-type, monoethenoid-type, and other types based on the parent structure.

#### 2.2.1. Diethenoid-Type Iridoids

**Figure 2:**
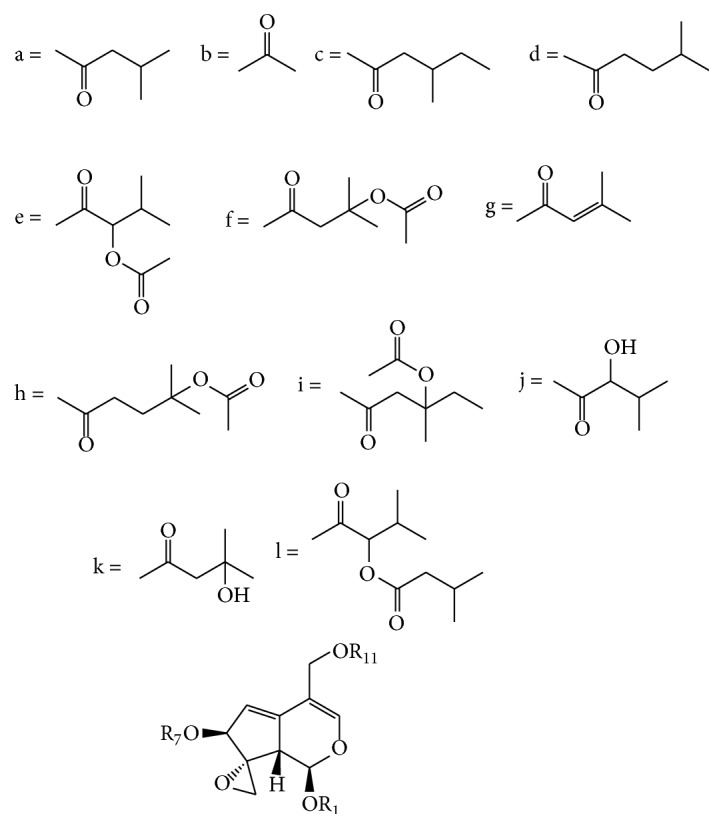
Compounds of diethenoid epoxy-type iridoids from* Valeriana* spp. (see Table 2).

**Figure 3:**
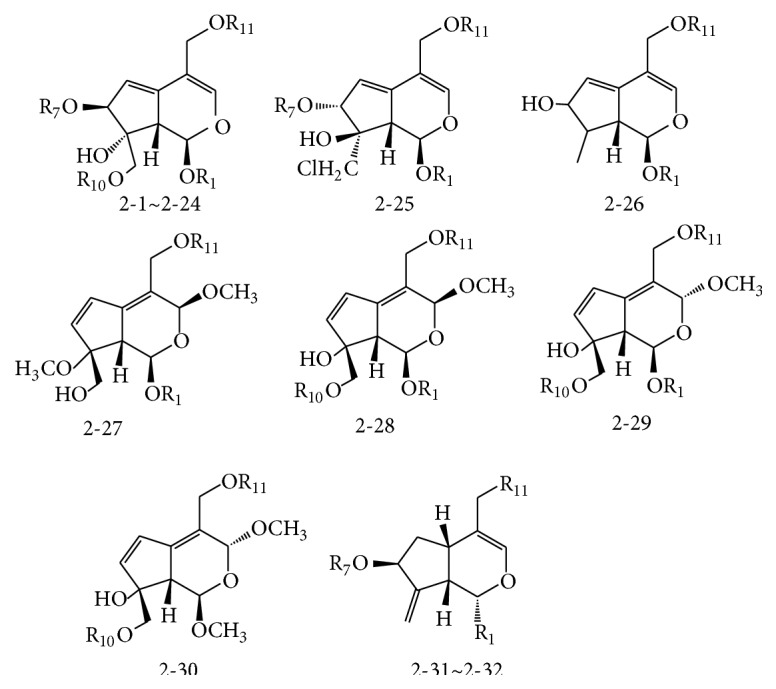
Compounds of diethenoid open ring-type iridoids from* Valeriana* spp. (see Table 3).

Diethenoid-type iridoids were characterized by the following molecular structures: (1) two C-C double bonds often presented between C-3 and C-4, C-5, and C-6 and occasionally presented between C-4 and C-5, C-6, and C-7; (2) an oxacyclopropane was often presented between C-8 and C-10, and C-10 was usually in a *β*-configuration; (3) H-1 (*α*-configuration) and H-9 (*β*-configuration) were preferentially located on different sides of the ring, and the C-7 acyl group usually was determined to be in the *β*-configuration (Figure 2 and Table 2 and Figure 3 and Table 3).

#### 2.2.2. Monoethenoid-Type Iridoids

**Figure 4:**
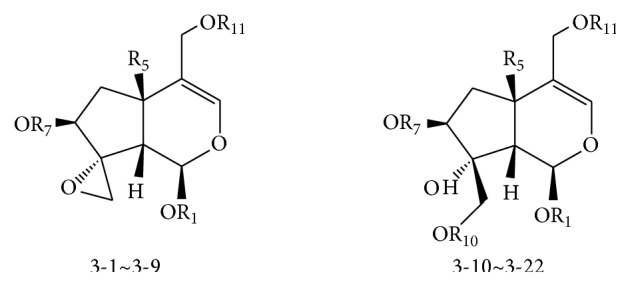
Compounds of monoethenoid-type iridoids from* Valeriana* spp. (see Table 4).

Monoethenoid-type iridoids were predominantly aglycones, which are characterized by the following structures: (1) a carbon double bond occurring mostly between C-3 and C-4; (2) H-1 (*α*-configuration), H-5 or 5-OH (*β*-configuration), H-7 (*β*-configuration), and H-9 (*β*-configuration); (3) a triatomic heterocyclic structure occurring between C-8 and C-10, and C-10 is usually a *β*-methylene, which is called monoene closed-loop iridoids. When C-8 and C-10 were not in ring formation, the structure is classified as a monoene open-loop iridoid (Figure 4 and Table 4).

#### 2.2.3. Other Types of Iridoids

**Figure 5:**
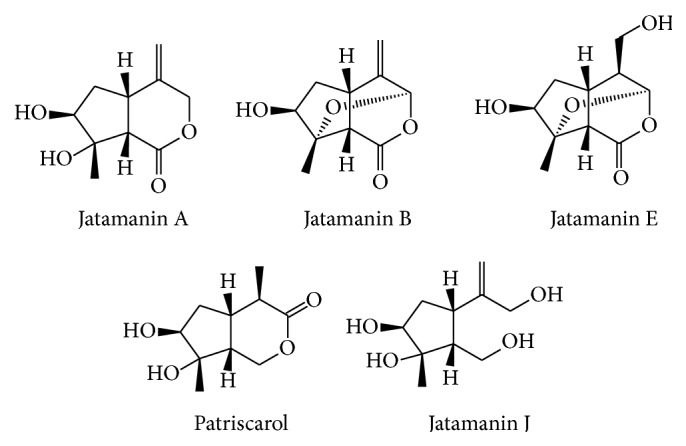
Compounds of other types of iridoids from* Valeriana* spp.

Iridoids from* Valeriana* spp. were mostly of the two above-mentioned types (Figure 5). In addition, other types were also identified: (1) most of one type having free hydroxyl groups and ester groups, with a lactone structure between C-1 and C-3 and a double bond between C-4 and C-1, (2) an oxygen bridge between C-3 and C-8, C-3 and C-10, or C-8 and C-11, (3) cleaved Ring-A of other types forming a free hydroxyl or aldehyde group between C-1 and C-3 (e.g., see Lin et al. [31]).

### 2.3. Lignanoids

Recent researches have indicated that lignanoids are 7,9-monoepoxy lignin and a glycoside or bisepoxy lignin. Britta Schumacher isolated eight lignanoids from* Valeriana officinalis*, namely, pinoresinol-4-O-D-glucoside, lignans 8′-hydro-xypinoresinol, 7,9′-monoepoxylignans massoniresinol-4′-O-beta-D-glucoside, berchemol-4′-O-D-glucoside, 8′-hydroxy-pinoresinol-4′-O-D-glucoside, and 8-hydroxypinoresinol-4′-O-D-glucoside [32]. Piccinelli et al. isolated two novel lignan glycosides from* Valeriana prionophylla*, including fraxireslnol-4′-O-D-glucopyranoside and prinse-piol-4-O-D-glucopy-ranoside [33].

### 2.4. Alkaloids

Alkaloids in* Valeriana* spp. included chatinine, nordelporphine, norphoebine, thaliperphine, nantenine, phenanthrene, phoebine, dehydroaphine, valerine, valeriane, and oxoaporphine, which occupy the low level of 1% [3, 34].

### 2.5. Flavonoids

Flavonoids in* Valeriana* spp. were mainly acacetin, apigenin, diosmetin, luteolin, quercetin, kaempferol, linarin, and luteolin [10, 33, 34], which occurred at low levels.

### 2.6. Amino Acids

Free amino acids in the water extracts of* Valeriana* spp. included *γ*-amino butyric acid (GABA), tyrosine, refined ammonia acid, glutamine, caffeic acid, chlorogenic acid, tannins, and sitosterol. GABA, a well-studied inhibitory neurotransmitter, is involved in lots of metabolic activities [59–62].

## 3. Research Advances on the Cardiovascular Activities of* Valeriana*

### 3.1. Reduction in Blood Pressure Level and Heart Rate

The increase of peripheral resistance in blood circulation was the common characteristic for primary hypertension, whose pathological mechanism was related to an increase of peripheral vascular tone and structural change of vessel walls. Additionally, structure and function disorders of vascular smooth muscle cells (VSMC) also contributed largely to this abnormal change. Therefore, improving the contract status of VSMC, expending the peripheral vessels, and inhibiting abnormal growth of VSMC preventing or alleviating vessel reconstruction at the same time were the keys to treating hypertension. Wang et al. [63] cultured aortic medial smooth muscle cells from a 6-month-old aborted fetus and examined the migration of cultured cells by Boyden Chamber. They found essential oil (VOL) could significantly inhibit the migration of human VSMC in a dose-dependent manner. Yang et al. [64] observed the effect of VOL and L-nitro arginine methyl ester (L-NAME) on the contraction of VSMC through the analogous experiment and investigated changes of ^3^H-thymidine (^3^H-TdR) and ^3^H-Leucine caused by angiotensin II (Ang II) and different concentrations of VSMC. VOL markedly inhibited the Ang II-stimulated contraction and growth of VSMC, which was not affected by L-NAME. In addition, VOL inhibited the incorporation of ^3^H-TdR and ^3^H-leucine. Zhou et al. [65] found that VOL could decrease the heart rate and blood pressure (priority to diastolic pressure) of rabbit and prolonged the duration of ST segment and T wave in a dose-dependent manner. VOL could decrease heart rate and blood pressure stimulated by adrenaline, which might be related to relaxing VSMC, enlarging vessel diameter, and decreasing blood resistance. VOL also observably inhibited contraction of VSMC stimulated by adrenaline, dilated coronary arteries, and decreased myocardial oxygen consumption. The vasorelaxant effects of the EtOH extract (1 mg·mL^−1^) and 8-hydroxypinoresinol (100 *µ*m) from the roots of* Valeriana prionophylla* have been already shown [66]. Fields et al. [66] reported that VOL could dilate pulmonary vessels in felines via a nonselective GABA mechanism and inhibited contraction of isolated frog hearts stimulated by cardenolide. It has already been shown that hexanic extracts (HEVe) from* V. edulis* ssp.* procera* enriched in valepotriates present vasorelaxant properties by blocking calcium channels. HEVe induced a significant concentration-dependent and endothelium-independent relaxation on isolated rat aorta precontracted with noradrenaline (0.1 *µ*m). HEVe, the most potent extract (0.15–50 *µ*g/mL), induced relaxation in aortic rings precontracted with KCl (80 mm), with IC_50_ value of 34.61 *µ*g/mL and *E*_max_ value of 85.0% [67].

### 3.2. Antimyocardial Ischemia Reperfusion Injury

As early as the 1980s, Zhang et al. [68] reported that the ethanol extract of valerian could dilate the coronary artery and reduce myocardial oxygen consumption in anesthetized cats. Yang et al. [69] reported that its essential oil and iridoids enhance microcirculation perfusion of the heart and kidney. The valerian extract can prevent injuries to myocardial ischemia reperfusion model in the rabbit by decreasing the levels of xanthine oxidase (XOD), malondialdehyde (MDA), and tumor necrosis factor-*α* (TNF-*α*), thereby increasing the 6-keto-prostaglandin F1*α*/thromboxane B2 (6-keto-PGF1*α*/TXB2) ratio. Huang et al. [70] conducted a study to investigate myocardial protection mechanism of monoterpene oxide of valerian (VMO). Compared to the control group, VMO showed a maximum change rate of left ventricular pressure, with a maximal rate of the increase of the left ventricular pressure (+*d*_ip_/*t*_max_) and maximal rate of the decrease of the left ventricular pressure (−*d*_ip_/*t*_max_) by 25.1% and 25.3%, respectively. Adenosine triphosphate (ATP) and energy charge (*E*_*C*_) increased by 72.8% and 20.9%, respectively, whereas myocardial creatine kinase-myocardial band (CK-MB) decreased by 20.7%. These results demonstrated the analogical performance between VMO and ischemic preconditioning pretreatment on cardio protection, which indicated a mobilizing myocardial endogenesis protective mechanism and an exoteric ATP-sensitive potassium channel. Yang et al. [71] set up an isolated rat ischemia reperfusion (I/R) heart model using a Langendorff-perfusion system, observing the effects of VOL pretreatment on I/R injury and related biochemical factors and cytosolic free calcium. The results indicated that VOL pretreatment markedly prevented I/R injury, weakened vasospasm perfusion, sustained the heartbeat, and reduced ventricular arrhythmic events in a dose-dependent manner. Simultaneously, VOL significantly lowered lactate dehydrogenase (LDH), creatine phosphokinase (CK), and MDA levels. The activities of superoxide dismutase (SOD), adenosine triphosphatase (ATPase), and glutathione peroxidase (GSH-Px) were enhanced. VOL reduced intracellular calcium in a concentration-dependent manner. The mechanism of action for VOL's aforementioned activities potentially involved preventing the increase in concentration of free Ca^2+^ and decrease in lipid peroxidation.

### 3.3. Antiarrhythmia

Arrhythmia is a common disease that involves various pathological mechanisms. Although western medicines have considerable efficiency, the adverse reactions at different levels and the development of arrhythmia caused by the drug itself have been reported. Therefore, it is imperative to discover an antiarrhythmic drug that features efficiency, stability, and the absence of adverse effects; these properties are inherent to traditional Chinese medicine, which are also of scientific and societal significance.

Arrhythmia induced by aconitine might be caused by myocardium excitability, which opens Na^+^ channel of the cardiac muscle and promotes sodium currents, resulting in a ventricular and supraventricular ectopic rhythm and ventricular tachycardia. Ventricular fibrillation induced by chloroform could be related to the release of neurotransmitters or adrenaline secretion in the adrenal medulla, as well as stimulation of *β* receptors [72, 73].

Jia and Zhang [74] found that chloroform extract of ethanol extract (v3d) could effectively prevent atrial fibrillation in mice induced by acetylcholine-calcium chloride and ventricular fibrillation induced by chloroform. It also protected rats from ischemia arrhythmia induced by ligation of the left anterior descending coronary artery. In addition, it effectively prevents dog auricular and renal vessels contraction induced by high K^+^ levels. Therefore, v3d prevents arrhythmia in various animal species partly by inhibiting Ca^2+^ channel from opening, which was induced by high K^+^ level.

Huang [75] found valerian extract (monoterpene and sesquiterpene oxides from essential oils) could dose-dependently reduce the duration of an action potential and inhibit Na current (*I*_Na_), L-type calcium current (*I*_Ca-L_), and transient outward potassium current (*I*_to_). It interacts with inactivated *I*_Na_ and *I*_Ca-L_, although various concentrations of v3d had no detectable effect on the delayed rectifier potassium current (*I*_K_) or inward rectifier potassium current (*I*_KI_) or direct interference with adenosine triphosphate sensitive potassium current (*I*_KATP_). The impacts of the valerian extract on these ion pathways might have contributed to its antiarrhythmia activity.

Wen et al. [76] reported the water, essential oil, and other fractions of valerian could protect a rat model from arrhythmia caused by aconitine or chloroform. Water extract at a dose of 50 and 25 g·kg^−1^ (calculated as raw herb) effectively decreased the occurrence of ventricular fibrillation, delayed the occurrence of arrhythmia, and decreased the mortality rate. The essential oil at a dose of 50 and 25 g·kg^−1^ (calculated as raw herb) effectively inhibited arrhythmia that was induced by chloroform; other fractions also demonstrated antiarrhythmia activities at different levels. Duan [77] found two active compounds from* V. officinalis* L., prinsepiol-4-O-*β*-D-glucoside and 8-hydroxy pinoresinol-4-O-*β*-D-glucoside; both showed antiarrhythmia activities. The former imparted an inhibitory effect on the Kv1.5 channel, which is the key mechanism for antiarrhythmia activity.

It was shown that didrovaltrate blocks L-type calcium current in a concentration-dependent manner and probably inhibited these currents in its inactive state. Didrovaltrate at concentrations of 30 *μ*g/L and 100 *μ*g/L significantly decreased peak *I*_Ca-L_ (*I*_Ca-Lmax_) from 6.01 to 3.45 pA/pF and 2.16 pA/pF, respectively. Didrovaltrate shifted upwards the current-voltage curves of *I*_Ca-L_ without changing their active, peak, and reverse potentials. Didrovaltrate affected the steady-state inactivation of *I*_Ca-L_. The half activation potential (*V*_1/2_) was significantly shifted from −26 to −36 mV, with a significant change in the slope factor (*k*) (from 8.8 to 11.1) [78].

Liu et al. [79] studied antiarrhythmia effective substances in serum of* V. officinalis* L. The study showed that borneol and bornyl acetate from Valerian essential oils and another unidentified compound from ethyl acetate extract could be absorbed into the blood in its original form, which indicated that this unidentified compound might be the main substance that contributes to the antiarrhythmia activity of the ethyl acetate extract.

### 3.4. Regulation of Blood Lipid Levels

Reports on* V. officinalis* L. var.* latifolia* Miq. (VOL) and its constituents in lipid regulation are limited. Hu et al. [80] examined the effects of VOL on blood lipid metabolism in rabbits with hyperlipidemia. VOL imparts a remarkable antilipid peroxidation effect, reduces the levels of serum total cholesterol (TC), triglyceride (TG), low-density lipoprotein cholesterol (LDL-C), and MDA, and elevates the levels of high-density lipoprotein cholesterol (HDL-C) and SOD. The results prove that it is imperative to further investigate the underlying mechanisms in regulating lipid metabolism. Si et al. [81] also demonstrated VOL could reduce the serum levels of total cholesterol, low-density lipoprotein, urinary albumin, and serum creatinine. Light microscopy and immunohistochemical stain revealed that, in the same time of lowering serum lipid, mesangial matrix index was significantly reduced, accompanied by decreased expression of TGF-*β*_1_ and type IV collagen.

## 4. Conclusions

*Valeriana* spp. possesses a wide range of bioactivities, which have been conferred by its complex and diverse active ingredients. Although the effects of* Valeriana* spp. mainly affected the cardiovascular system in Section 3, its mechanism of action needs to be further investigated.

## Figures and Tables

**Table 1:** The list of essential oil constituents from *V. officinalis *L. var.* latifolia *Miq.

Signal	Compounds	Molecular format	Molecular weight	Retention time (min)	Content (%)
1	Carene	C_10_H_16_	136	6.100	0.29
2	*α*-Thujene	C_10_H_16_	136	6.473	4.18
3	6-Isopropyl-1-methyl bicycles [3,1,0] hexane	C_10_H_16_	136	6.983	14.19
4	Sabinene	C_10_H_16_	136	7.736	2.55
5	p-Cymene	C_10_H_14_	134	9. 143	0.43
6	Limonene	C_10_H_16_	136	9.281	1.26
7	Camphor	C_10_H_16_O	152	12.596	0.19
8	Borneol	C_10_H_18_O	154	13.671	3.54
9	L-Myrtanol	C_11_H_16_O	164	14.584	0.81
10	*α*-Methyl 4(1′, 1′-methyl ethyl) phenol	C_11_H_16_O	164	15.432	2.49
11	Bornyl acetate	C_12_H_20_O_2_	196	17.311	23.93
12	Sabinol	C_10_H_16_O	152	18.270	1.70
13	*α*-Terpineol	C_10_H_18_O	154	18.944	1.20
14	*β*-Caryophyllene	C_15_H_24_	204	20.989	0.82
15	*β*-Gurjunene	C_15_H_24_	204	21.343	1.16
16	Humulene	C_15_H_24_	204	21.891	0.40
17	Unidentified	C_15_H_22_	202	22.045	1.32
18	*trans*-caryophyllene	C_15_H_24_	204	22.450	0.28
19	Nerolidol	C_15_H_24_O	220	22.838	0.78
20	Elemene	C_15_H_24_	204	22.977	0.45
21	Bornyl isovalerianate	C_15_H_26_O	238	23.441	0.36
22	Azulene furan	C_15_H_10_O_2_	222	23.867	0.58
23	Stereoisomer of ramie enol	C_10_H_16_O	152	24.485	1.46
24	4a,8-Dimethyl-*α*-isopropyl naphthyl ketone	C_15_H_24_O	220	25.155	2.77
25	Tetramethyl-4-hydroxyl cyclopropane naphthalene	C_15_H_24_O	220	25.294	1.26
26	Unidentified	C_15_H_24_O	220	25.790	1.72
27	Ledol	C_15_H_26_O	222	27.011	1.22
28	Guaiol	C_15_H_26_O	222	27.150	4.73
29	Valerone	C_15_H_26_O	222	27.493	1.14
30	Nootkatone	C_15_H_22_O	218	29.031	14.79
31	Nootkatone isomer 1	C_15_H_22_O	218	29.467	1.06
32	Nootkatone isomer 2	C_15_H_22_O	218	30.333	0.90
33	1,2,3,4,4a,5,6,8,8a-Eight hydrogen-4a,8-dimethyl-*α*-Propenyl [*α*] naphthyl alcohol	C_15_H_24_O	220	35.174	0.83
34	Unidentified			36.500	0.83

**Table 2:** 

Number	R_1_	R_7_	R_11_	Compounds	References
1-1	a	a	b	Valtrate	[6]
1-2	a	f	b	Acevaltrate	[6]
1-3	a	b	a	Isovaltrate	[35]
1-4	a	H	b	7-Epi-deacetylisovaltrate	[36]
1-5	a	H	a	Deacetylisovaltrate	[37]
1-6	d	a	b	Homovaltrate 1	[35]
1-7	a	b	d	Homovaltrate 2	[35]
1-8	f	a	b	1-*β*-Acevaltratum	[38]
1-9	g	a	b	1-Seneciovaltrate	[39]
1-10	a	b	b	Diavaltrate	[40]
1-11	f	f	b	1-*β*-Aceacevaltrate	[40]
1-12	c	b	a	Homoisovaltrate	[41]
1-13	c	c	b	1,7-Dihomovaltrate	[41]
1-14	e	a	b	1-*α*-Acevaltrate	[41]
1-15	e	c	b	Homo-A	[41]
1-16	c	b	b	Homo-B	[41]
1-17	a	c	k	Homo-Z	[41]
1-18	e	b	a	1-*α*-Aceisovaltrate	[42]
1-19	a	c	b	Homovaltrate	[43]
1-20	c	f	b	1-Homovaltrate	[44]
1-21	c	b	e	1-Homoisoacevaltrate	[44]
1-22	g	a	a	Sorbifolivaltrate A	[45]
1-23	g	c	b	Sorbifolivaltrate B	[45]
1-24	a	a	H	Deacetlyvaltrate	[46]
1-25	a	c	b	7-Homovaltrate	[46]
1-26	c	a	b	1-Homovaltrate	[46]
1-27	a	b	e	11-Acevaltrate	[46]
1-28	a	i	b	Homoacevaltrate	[46]
1-29	a	k	b	Hydroxyvaltrate	[47]
1-30	a	h	b	Isohomovaltrate	[47]

**Table 3:** 

Number	R_1_	R_7_	R_10_	R_11_	Compounds	References
2-1	a	a	a	b	Valtrate-isovaleroxyhydrin	[37]
2-2	a	a	a	b	Valtrate hydrin B1	[47]
2-3	a	a	b	b	Valtrate hydrin B2	[47]
2-4	j	a	a	b	Valtrate hydrin B3	[47]
2-5	a	b	a	a	Valtrate hydrin B4	[39]
2-6	a	a	c	b	Valtrate hydrin B5	[39]
2-7	a	b	c	b	Valtrate hydrin B6	[39]
2-8	g	a	a	b	Valtrate hydrin B7	[39]
2-9	e	a	a	b	Valtrate hydrin B8	[39]
2-10	e	b	b	a	Acetoxydesiovaleroxy-1-*α*-acetoxy-isovaleroxy isovaltratehydrine	[42]
2-11	c	a	b	b	10-Acetoxy-1-homovaltrate hydrin	[44]
2-12	f	a	b	b	10-Acetoxy-1-acevaltrate hydrin	[44]
2-13	k	a	a	a	Sorbifolivaltrate C	[45]
2-14	g	c	a	b	Sorbifolivaltrate D	[45]
2-15	a	e	l	b	Valeriandoid F	[48]
2-16	a	b	X	a	Jatamanvaltrate I	[49]
2-17	a	H	a	b	Jatamanvaltrate J	[49]
2-18	a	a	H	a	Jatamanvaltrate K	[49]
2-19	a	a	b	b	10-Acetoxyvaltrahedrin	[49]
2-20	a	b	Cl	a	Rupesin B	[50]
2-21	b	H	Cl	a	Valeriandoids A	[51]
2-22	a	f	Cl	b	Valeriandoids B	[51]
2-23	a	b	a	a	Isovaltrate isovaleroyloxyhydrin	[51]
2-24	a	a	Me	b	Valeriandoids F	[52]
2-25	a	a	—	b	Volechlorine	[36]
2-26	a	—	—	a	Nardostachin	[53]
2-27	a	—	—	b	Jatamanvaltrate N	[50]
2-28	a	—	l	b	Jatamanvaltrate O	[50]
2-29	a	—	l	b	Valeriandoids D	[52]
2-30	—	—	l	b	Valeriandoids E	[52]
2-31	a	b	—	a	8,11-Desoidodidrovaltrate	[37]
2-32	d	b	—	a	8,11-Desoidohomoddidrovaltrate	[37]

**Table 4:** 

Number	R_1_	R_5_	R_7_	R_10_	R_11_	Compounds	References
3-1	a	H	a	—	b	Didrovaltrate	[6, 37, 53]
3-2	a	OH	b	—	l	Isovaleroxyhydroxydihydrovaltrate	[43]
3-3	a	H	b	—	a	Isodidrovaltrate	[54]
3-4	a	OH	b	—	e	AHD-valtrate	[40]
3-5	a	OH	b	—	c	11-Homohydroxyldihydrovaltrate	[44]
3-6	c	H	b	—	a	Homodidrovaltrate	[37]
3-7	a	OH	H	—	l	Jatamanvaltrate L	[49]
3-8	a	OH	b	—	Et	Jatamanvaltrate M	[49]
3-9	a	OH	b	—	a	5-Hydroxydidrovaltrate	[49]
3-10	a	OH	H	b	l	Valeriotriate B	[55]
3-11	a	OH	b	l	l	Valeriotertrate A	[56]
3-12	a	OH	b	f	l	Jatamanvaltrate A	[49]
3-13	a	OH	b	a	l	Jatamanvaltrate B	[49]
3-14	a	OH	b	b	l	Jatamanvaltrate C	[49]
3-15	a	OH	b	X	l	Jatamanvaltrate D	[49]
3-16	a	OH	b	Me	l	Jatamanvaltrate E	[49]
3-17	a	H	b	f	a	Jatamanvaltrate F	[49]
3-18	a	H	H	b	a	Jatamanvaltrate J	[49]
3-19	a	H	b	H	a	Jatamanvaltrate K	[49]
3-20	a	H	b	b	a	Didrovaltrate acetoxyhydrin	[49]
3-21	a	OH	b	Cl	l	Volvatrate B	[57]
3-22	a	OH	H	Cl	l	Jatamandoid A	[58]

## Abbreviations

+*d*_ip_/*t*_max_: Maximal rate of the increase of the left ventricular pressure
^3^H-TdR: ^3^H-Thymidine
6-keto-PGF1*α*/TXB2: 6-Keto-prostaglandin F1*α*/thromboxane B2
Ang II: Angiotensin II
ATP: Adenosine triphosphate
CK: Creatine phosphokinase
CK-MB: Creatine kinase-myocardial band
−*d*_ip_/*t*_max_: Maximal rate of the decrease of the left ventricular pressure
*E*_*C*_: Energy charge
GABA: *γ*-Amino butyric acid
GC-MS: Chromatography-mass spectrometry
GSH-Px: Glutathione peroxidase
HDL-C: High-density lipoprotein cholesterol
HEVe: Hexanic extracts
I/R: Ischemia reperfusion
*I*_Ca-L_: L-type calcium current
*I*_K_: Delayed rectifier potassium current
*I*_KATP_: Adenosine triphosphate sensitive potassium current
*I*_KI_: Inward rectifier potassium current
*I*_Na_: Na current
*I*_to_: Transient outward potassium current
LDH: Lactate dehydrogenase
LDL-C: Low-density lipoprotein cholesterol
L-NAME: L-nitro arginine methyl ester
MDA: Malondialdehyde
RP-HPLC: Reverse phase high-performance liquid chromatography
SOD: Superoxide dismutase
TC: Total cholesterol
TG: Triglyceride
TNF-*α*: Tumor necrosis factor-*α*
v3d: Chloroform extract of ethanol extract
VMO: Monoterpene oxide of valerian
VOL: Essential oil
VSMC: Vascular smooth muscle cells
XOD: Xanthine oxidase.
